# Diversity and Community Composition of Vertebrates in Desert River Habitats

**DOI:** 10.1371/journal.pone.0144258

**Published:** 2015-12-04

**Authors:** C. L. Free, G. S. Baxter, C. R. Dickman, A. Lisle, L. K.-P. Leung

**Affiliations:** 1 School of Agriculture and Food Sciences, University of Queensland, Gatton, Queensland 4343, Australia; 2 School of Geography, Planning and Environmental Management, University of Queensland, St Lucia, Queensland 4072, Australia; 3 Desert Ecology Research Group, School of Biological Sciences, University of Sydney, Sydney, New South Wales 2006, Australia; University of Arkansas, UNITED STATES

## Abstract

Animal species are seldom distributed evenly at either local or larger spatial scales, and instead tend to aggregate in sites that meet their resource requirements and maximise fitness. This tendency is likely to be especially marked in arid regions where species could be expected to concentrate at resource-rich oases. In this study, we first test the hypothesis that productive riparian sites in arid Australia support higher vertebrate diversity than other desert habitats, and then elucidate the habitats selected by different species. We addressed the first aim by examining the diversity and composition of vertebrate assemblages inhabiting the Field River and adjacent sand dunes in the Simpson Desert, western Queensland, over a period of two and a half years. The second aim was addressed by examining species composition in riparian and sand dune habitats in dry and wet years. Vertebrate species richness was estimated to be highest (54 species) in the riverine habitats and lowest on the surrounding dune habitats (45 species). The riverine habitats had different species pools compared to the dune habitats. Several species, including the agamid *Gowidon longirostris* and tree frog *Litoria rubella*, inhabited the riverine habitats exclusively, while others such as the skinks *Ctenotus ariadnae* and *C*. *dux* were captured only in the dune habitats. The results suggest that, on a local scale, diversity is higher along riparian corridors and that riparian woodland is important for tree-dependent species. Further, the distribution of some species, such as *Mus musculus*, may be governed by environmental variables (e.g. soil moisture) associated with riparian corridors that are not available in the surrounding desert environment. We conclude that inland river systems may be often of high conservation value, and that management should be initiated where possible to alleviate threats to their continued functioning.

## Introduction

Biodiversity does not occur evenly across space or time [1], with species often occurring patchily at multiple scales. Environmental variables such as climate, habitat, water and food influence diversity in a given area or at a given time, with additional effects being wrought by interactions among individual species [2, 3]. Scientists and land managers invest much time and many resources in trying to identify and conserve those habitats and areas that have high species richness, diversity and levels of endemism. In turn, national and international agreements and legislation (e.g. Convention of Biological Diversity 1993, *Environment Protection and Biodiversity Conservation Act* 1999) aim to ensure conservation of areas that support high biodiversity on both national and international scales.

Riparian corridors often contain higher levels of biodiversity than their surrounding catchments [4–7]. At one level, this is not surprising: fish, freshwater invertebrates and other species that require continuous access to free water will occur in the riverine system but not in the drier areas beyond [8, 9]. However, high riparian diversity may be observed even in ephemeral systems where the flow of water is too low or discontinuous to sustain species that require free water [7]. This pattern could be expected to be most pronounced in arid areas where differences between the riparian corridor and adjacent habitat are most marked [4].

Three processes potentially elevate levels of biodiversity along ephemeral arid rivers. Firstly, riparian corridors that originate in high elevation or high rainfall areas outside the desert could act as conduits for mesic-adapted (peri-desert) biota, allowing them to penetrate deep into arid regions. This effect is most obvious for broad-leaved or non-sclerophyllous plants that cannot tolerate extreme water stress [10, 11], and for mobile organisms such as bats and birds that track the pulses of productivity that follow transient river flows [5, 12]. It may hold also for less mobile organisms, although evidence for this is more limited [13]. Secondly, corridors may act as drought refugia for desert biota when conditions in the surrounding environment become too dry and unproductive to support them. Large mammals such as Giraffe (*Giraffa camelopardalis*) and the Red Kangaroo (*Osphranter rufus*), as well as large birds such as Emus (*Dromaius novaehollandiae*), regularly exhibit such shifts into riparian areas during drought [14–16]. Several species of medium-sized Australian desert marsupials are suspected also to have contracted to regional refugia during drought, although few now remain owing to the degradation of these refugia since European settlement [17, 18]. Thirdly, some biota may be endemic to arid riparian corridors. Such species could be expected in long-established desert systems, such as the Namib or parts of arid Australia [8, 19], and to show affinities to taxa either in the adjacent desert or in fringing mesic areas [20].

In this study, we tested the general hypothesis that riparian sites in arid environments support higher vertebrate diversity than other desert habitats. This hypothesis is tested on small terrestrial species rather than on large mammals or birds, as the latter groups have been subject to considerable study [5, 16]. Following support of the general hypothesis, we tested three specific but alternative hypotheses about the processes that generate high vertebrate diversity along desert river systems:

1. If desert river corridors allow ingress of species from non-desert regions, species richness along corridors should decline from the desert fringes to the interior.
2. If these corridors act as drought refugia, more species should be found along corridors during dry periods than after rain.
3. If these corridors harbour endemic biota, there should be differences in species composition between corridor and other habitats, with some species found uniquely along the corridors.

To test these hypotheses, and to investigate species-habitat associations, distributional and compositional data were collected over two and a half years on the vertebrate species inhabiting a riverine corridor and adjacent habitats at a site in arid Australia. The site, along the Field River in the Simpson Desert of western Queensland, was selected for several reasons. Firstly, the habitat characteristics of the Field River vary along its length and from the riparian corridor to the arid sand dune environment through which the river runs. The corridor has different soil properties and greater soil moisture, soil nutrients and cover of trees, grasses, annual herbs and leaf litter than the surrounding dunes, and thus has higher primary productivity [21]. Secondly, the study region experiences highly irregular rainfall, allowing us to sample during both dry and wet periods. The Field River is, in consequence, ephemeral, running only after on-site rain or rainfall in the broader catchment. Thirdly, the river is likely to be long-established; the Simpson Desert formed around two million years ago (although the dune fields are more recent), and the river would have experienced flows even during dry periods owing to its catchment in the southern part of the monsoonal tropics [22]. We comment, finally, on the conservation implications of our findings.

## Materials and Methods

### Study sites and climate

This study was conducted along the Field River (S 23°48’ E 138°03’) on Ethabuka Reserve (S 23°46’ E 138°28’) in the north-eastern part of the Simpson Desert, around 200 km west-south-west of Boulia, Queensland. The reserve is located in the Simpson-Strzelecki Bioregion [23] and is dominated by long parallel sand dunes that run in a NNW–SSE direction [24]. The reserve was used for cattle grazing until 2004, but was de-stocked in that year and is now leased by Bush Heritage Australia as a conservation reserve.

The Field River originates in the Toko Ranges on the Queensland/Northern Territory border [25]. Flows are sometimes driven by heavy on-site rainfall, but most usually by rainfall in the Toko catchment. This catchment lies on the northern fringe of the Simpson Desert at the southern extremity of the northern monsoonal tropics [22]. Along the river the dominant vegetation includes *Corymbia terminalis*, *Eucalyptus camaldulensis*, *E*. *coolabah*, *Eremophila* spp. and *Eulalia aurea*. The dominant vegetation in the surrounding dune fields is the hummock grass *Triodia basedowii* (hard spinifex), interspersed with perennial shrubs such as *Acacia* spp., *Grevillea* spp., *Eremophila* spp., *Sida* spp., *Dicrastylis* spp. and *Crotalaria* spp. [26, 27]; annual grasses and herbs are abundant after rain.

The Simpson Desert is a hot sandy desert and lies within the 150 mm rainfall isopleth. The Field River catchment received an average of 152 mm of rainfall annually from 1995 to 2008 (recorded from two weather stations [Environdata, Warwick, Queensland]—Field River South and Field River North—located between sites established along the river channel for the present study). During the study period (2006 to 2008), monthly rainfall was generally below average, but significant (337 mm) rainfall was recorded in January 2007 at both weather stations. During the course of the study the monthly maximum summer temperatures along the Field River ranged from 39°C to 48°C, with minimum winter temperatures from -5°C to 1.3°C.

### Vertebrate sampling

Three pitfall trapping sites were established along the Field River to sample the diversity of small vertebrates at different stages along the riparian system. The first site (Field River South) was established on the southern border of the reserve (S 23° 58’ 19.3” E 138° 08’ 31.2”), the second (Field River Middle) was 20 km to the north and the final (Field River North) was established another 20 km north. The latter site was some 80 km south of the main catchment area for the river [25].

**Fig 1:**
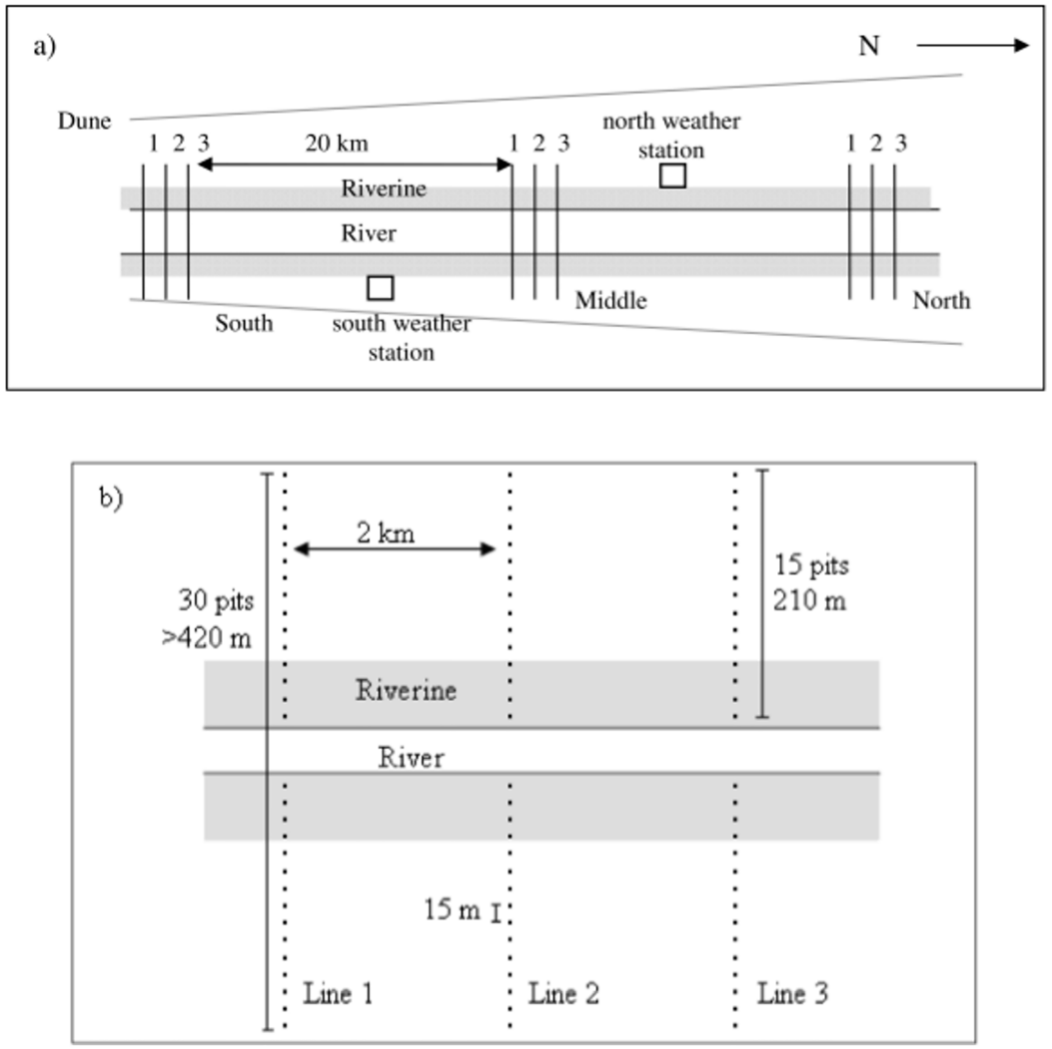
Schematic layout of pitfall traps used to sample small vertebrates along the Field River, Simpson Desert, western Queensland. a) location of the three trapping sites along the Field River showing the distance between sites and the three pitfall transects at each site (numbered 1, 2 and 3), b) layout of traps within a site showing distance between pitfall transects and individual traps. Each dot represents one pit. Shaded areas represent the riverine corridor. Figure not to scale.

At each site, in October 2005, three parallel transect lines of pitfall traps were established, each spaced 2 km apart, that ran in a roughly east-west direction and thus intersected the river at 90°. Each transect consisted of 30 pits (Fig 1). Pits were placed along each transect, beginning beside the bank of the river and then every 15 m until there were 15 pits on each side of the river (Fig 1). This sampling design allowed stratification by vegetation, width of riverine habitat and distance from the northern ranges. Pitfall traps consisted of plastic (PVC) pipe (16 cm diameter, 60 cm deep) buried flush with the ground, with a fly wire base to prevent animals escaping. A 5 m-long (30 cm high) aluminium fly wire fence ran across the opening of each pit to increase trap efficiency. The use of pitfall trap transects followed previous research showing that this is the most efficient method of sampling small, ground-active vertebrates in arid Australian habitats [28, 29]. Pilot trials in the study region using potential alternative methods, such as folding metal traps, have yielded very few captures of any species [26, 27]; tracking and direct observations of small vertebrates also were not considered feasible owing to heavy vegetation cover in some habitats. The relatively narrow (16 cm diameter) pitfall traps that we used compared to wider traps (e.g. 28 cm diameter, [28]) that have been employed in some previous studies had one further advantage: they minimised captures of larger, predatory species such as large snakes and goannas (*Varanus* spp.) that could otherwise have preyed upon small mammals or lizards that had been captured earlier. We still captured 3–4 large individuals of these species over the course of the study and it is therefore possible that up to 3–4 smaller prey animals were consumed while in traps. There is currently no known way of entirely stopping predator species from accessing pitfall traps, and this methodology therefore remains the industry standard for efficiently surveying small, ground-active vertebrates. However, the narrow traps would have greatly reduced the risk of predator intrusion and, if a maximum of 3–4 prey individuals had been lost, we are confident that this would have produced little or no bias in our results.

Pits were opened for three consecutive nights in March, June and October 2006; May and September 2007; and March and May 2008. This allowed three sampling occasions during a dry period prior to the heavy rainfall event in January 2007 and four sampling occasions after. In March 2006 and September 2007, traps at the Field River Middle site were open for five nights. Due to local flooding, the northern site was not trapped in March 2006.

Captured animals were identified, weighed, measured and marked as part of additional studies of their population dynamics. Reptiles over 1 g were marked using a toe-clipping system, while mammals were given unique ear-clips. Frogs, snakes, legless lizards and lizards with reduced numbers of toes (*Lerista* spp.) were not marked. The immediate surroundings of traps were sprinkled lightly with Coopex^™^ ant powder to prevent ants from entering the traps [30]. Traps were closed with metal lids at the end of each trapping session.

This study was carried out in strict accordance with the recommendations of the Australian Code of Practice for the care and use of animals for scientific purposes. The protocol was approved by the University of Queensland Animal Ethics Committee (Approval number SAS/610/06/UQ/US). The study was conducted on private leasehold land with permission of the leaseholder (Bush Heritage Australia). The native animals trapped in the study were protected under the Queensland *Nature Conservation Act 1992* and a scientific purposes permit was required to conduct the study. A scientific purposes permit under the *Nature Conservation Act 1992* was granted by the Queensland Parks and Wildlife Service (permit WISP04088306) for the methods stated in this article.

### Habitat characteristics

**Fig 2:**
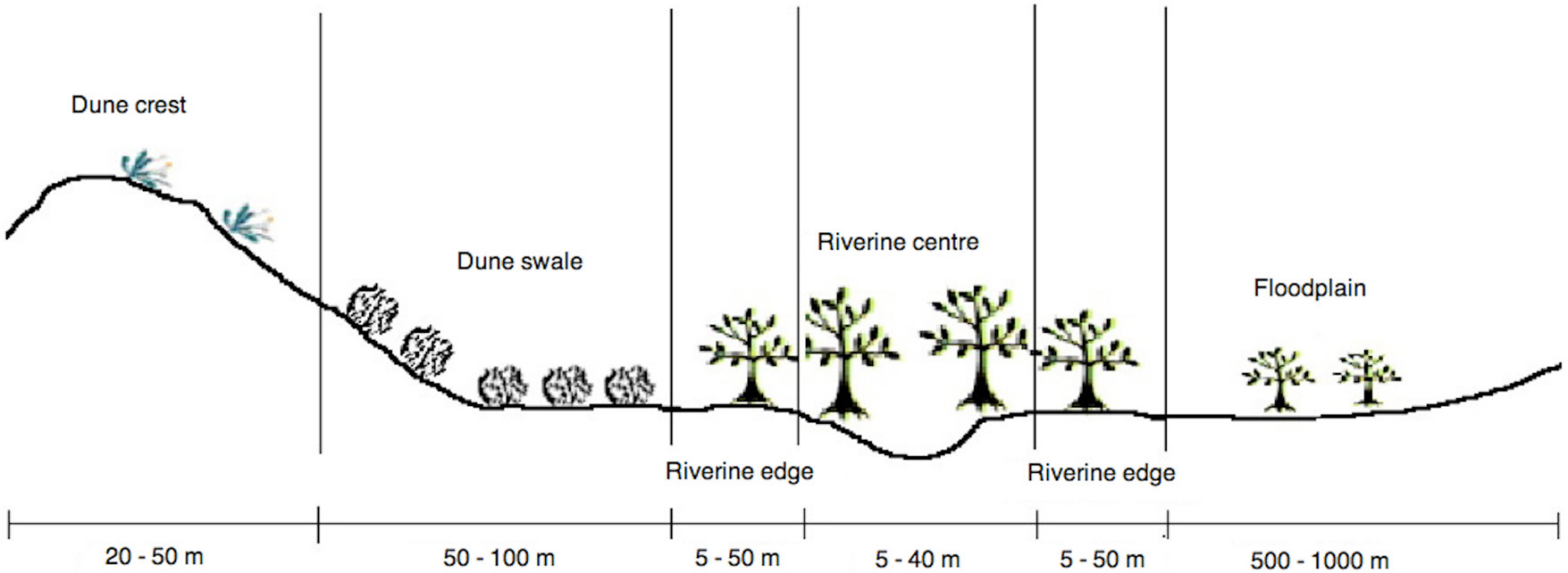
Cross section of the landscape along the Field River, Simpson Desert, western Queensland. This shows the range and approximate width of the five habitat types that were sampled: dune crest, dune swale, riverine edge, riverine centre and floodplain. The floodplain habitat was represented only in the northern study site.

Each pitfall trap was classified as being in one of five habitat types (Fig 2):

1. Dune crest: This comprised the top of a dune and the first few metres of the slope. The dominant vegetation usually consisted of *Acacia ligulata*, *A*. *dictyophleba*, *Grevillea stenobotrya*, *Zygochloa paradoxa*, *Dicrastylis costelloi* and *Sida* spp.
2. Dune swale: The dune swale was considered to run from the dune base to the dune slope (up to 2–4 m from the dune crest) with almost complete dominance by *Triodia basedowii*.
3. Riverine edge: This habitat was situated in the ecotone between the central riverine woodland and the dune swale. The vegetation in this habitat type was a mixture of *Triodia basedowii*, *Eucalyptus* spp., *Grevillea* spp. and *Acacia* spp.
4. Riverine centre: The riverine centre habitats were located on the immediate banks of the river and were dominated by tall trees including eucalypts (*Eucalyptus* spp.) and *Corymbia terminalis*. Native grasses (*Aristida contorta*, *Eulalia aurea*) were also present at most times.
5. Floodplain: Open clay floodplains occurred only in the northern study site. They were characterised by having little or no understorey with intermittent mallee shrubs dominated by *Eucalyptus gamophylla* and *E*. *pachyphylla*.

To investigate associations between vertebrates and finer-scale aspects of habitat, we sampled a range of environmental variables in circular plots (radius 2.5 m), centred around the pitfall traps at each of the sites on most sampling occasions. Sampling methods have been described in detail in [21] but, in brief, these were designed to estimate coverage of hard spinifex, annual grasses, other annual plants, shrubs, trees, leaf litter and other detritus, as well as plant species richness, distance to the nearest tree, soil moisture, and numbers of invertebrates captured in small (125 ml) plastic pitfall traps filled with 5% formalin solution [21]. These variables were considered to provide different types of cover, and food (invertebrates), that could be potentially used by most of the small vertebrates encountered during the study.

### Statistical analysis

Species diversity was expressed both as the actual numbers of species found in samples (species richness), and as estimates calculated using the first order jackknife algorithm in EstimateS [31]. The jackknife procedure was used to assess the numbers of species likely to have been missed during field sampling.

Data were pooled over the seven trapping sessions to give estimates over sites, habitats and pre- and post-rain. Capture rates at each sampling session were generally low (between 4% and 25%) and pooling the data allowed the hypotheses to be tested using the maximum number of individuals. Traps were repeatedly sampled over time, and this can cause auto-correlation of data; however, repeatedly trapping the same sites also should allow for increased accuracy in species richness estimation. Thompson and Withers [32] demonstrated that increased sampling effort is required to get accurate species richness estimates in areas where there is a high proportion of rare species in the community. During this study 55% of all species were rare (captured less than 10 times), so our repeated sampling of the trapping sites was expected to increase the number of individuals and accuracy of the species richness estimates. Further, all sites and transects were trapped at approximately the same time (over a 10–15 day period) at each sampling session, reducing the effect of time between sites.

Species composition between sites and habitats was compared using Bray-Curtis similarity indexes, as calculated in PRIMER-E [33], using non-transformed count data. Dendrograms were created using cluster ordination for each habitat type and site. The Simper procedure in PRIMER-E was used to calculate the contributions of each species to similarities between habitats and sites.

Canonical correspondence analysis (CCA), a form of gradient analysis, was used to examine the associations of individual species with environmental variables as described in [21]. In a CCA biplot, the importance of each environmental variable is indicated by the length of its associated arrow; variables with long arrows are more important in explaining the distribution of species than are variables with short arrows [34]. Arrows that are close together (that form an acute angle) are more highly correlated than those pointing in opposite directions, which have an inverse relationship [35, 36]. In CCA biplots, species that lie near the outside of the biplots are more strongly correlated with environmental variables than those that lie near the centre [35]. Because of low sample sizes for most species, only common species were plotted using CCA. These species were the Sandy Inland Mouse (*Pseudomys hermannsburgensis*), Central Netted Dragon (*Ctenophorus nuchalis*), Southern Sandslider (*Lerista labialis*), House Mouse (*Mus musculus*), Military Dragon (*Ctenophorus isolepis*), Eyre Basin Beaked Gecko (*Rhynchoedura eyrensis*), and Fire-tailed Skink *(Morethia ruficauda*). In addition, only those vertebrates captured and environmental variables sampled in spring 2006 (prior to the rain) and spring 2007 (seven months following the rain) were analysed in CCA plots, as these were the only occasions when large enough sample sizes were obtained for results to be reliable.

## Results

### Species diversity

In total, 1,386 individual vertebrates were captured over 5,638 pitfall trap nights. Vertebrates from 11 families were detected over all sampling occasions, with members of four families (Muridae, Scincidae, Agamidae and Myobatrachidae) being the most commonly trapped. The 11 families were represented by 46 species; skinks, with 14 species, were the most species-rich, followed by agamids and geckoes with six species each (Table 1).

**Table 1:** Distributions of small terrestrial vertebrate species in five different habitats along the Field River, western Queensland, from March 2006 to May 2008. Numbers represent numbers of individuals captured.

Family	Common Name	Dune crest	Dune swale	Flood-plain	Riverine centre	Riverine edge
**Hylidae**						
*Cyclorana cultripes*	Desert Collared Frog	0	1	0	0	2
*Litoria rubella*	Desert Tree Frog	0	0	1	1	3
**Myobatrachidae**						
*Platyplectrum spenceri*	Desert Burrowing Frog	0	0	0	3	2
*Neobatrachus sudellae*	Sudell's Frog	11	67	1	36	32
*Notaden nichollsi*	Spade-foot Toad	25	17	3	1	3
**Agamidae**						
*Gowidon longirostris*	Long-nosed Dragon	0	0	0	2	2
*Ctenophorus clayi*	Black-collared Dragon	5	6	1	1	0
*Ctenophorus isolepis* *	Military Dragon	6	24	5	15	2
*Ctenophorus nuchalis* *	Central Netted Dragon	16	19	23	19	16
*Pogona vitticeps*	Central Bearded Dragon	0	2	0	0	0
*Moloch horridus*	Thorny Devil	2	1	0	1	0
**Elapidae**						
*Pseudonaja nuchalis*	Western Brown Snake	0	0	2	0	0
**Diplodactylidae**						
*Diplodactylus conspicillatus*	Fat-tailed Gecko	0	6	0	0	0
*Lucasium stenodactylum*	Crowned Gecko	0	2	0	2	0
**Carphodactylidae**						
*Nephrurus levis*	Three-lined Knob-tail Gecko	0	3	0	3	3
**Gekkonidae**						
*Heteronotia binoei*	Bynoe's Gecko	1	3	1	2	2
*Gehrya variegata*	Tree Dtella	0	0	0	0	1
*Rhynchoedura eyrensis* *	Eyre Basin Beaked Gecko	4	4	9	32	13
**Pygopodidae**						
*Delma tincta*	Excitable Delma	0	0	1	5	1
*Lialis burtonis*	Burton's Legless Lizard	0	0	0	0	2
*Pygopus nigriceps*	Western Hooded Scaly-foot	0	0	0	1	0
**Scincidae**						
*Ctenotus ariadnae*	Ariadna's Ctenotus	3	9	0	0	0
*Ctenotus calurus*	Blue-tailed Ctenotus	1	3	0	0	0
*Ctenotus dux*	Chief Ctenotus	5	1	0	0	0
*Ctenotus helenae*	Helen's Ctenotus	0	6	4	12	6
*Ctenotus leae*	Lea's Ctenotus	2	0	0	0	0
*Ctenotus pantherinus*	Leopard Ctenotus	1	12	0	1	2
*Ctenotus piankai*	Pianka's Ctenotus	1	0	1	1	0
*Ctenotus regius*	Royal Ctenotus	0	1	1	1	0
*Liopholis inornata*	Desert Skink	0	3	0	5	0
*Lerista aericeps*	Desert Plain Slider	0	2	5	1	3
*Lerista labialis* *	Southern Sandslider	45	32	15	31	19
*Menetia greyi*	Common Dwarf Skink	0	4	0	1	0
*Menetia maini*	Northern Dwarf Skink	0	3	6	7	6
*Morethia ruficauda* *	Fire-tailed Skink	0	5	7	9	13
**Typhlopidae**						
*Ramphotyphlops endoterus*	Interior Blind Snake	3	5	0	2	2
**Varanidae**						
*Varanus brevicaudus*	Short-tailed Pygmy Monitor	3	4	0	1	1
*Varanus eremius*	Pygmy Desert Monitor	0	2	0	3	0
*Varanus gilleni*	Pygmy Mulga Monitor	0	1	0	2	0
*Varanus gouldii*	Sand Monitor	2	6	1	3	5
**Dasyuridae**						
*Ningaui ridei*	Wongai Ningaui	1	8	0	3	0
*Sminthopsis hirtipes*	Hairy-footed Dunnart	3	5	1	1	1
*Sminthopsis youngsoni*	Lesser Hairy-footed Dunnart	7	10	8	5	2
**Muridae**						
*Mus musculus#*	House Mouse	1	10	39	23	19
*Notomys alexis*	Spinifex Hopping-mouse	3	2	0	1	0
*Pseudomys hermannsburgensis* *	Sandy Inland Mouse	33	103	97	85	65
	Total	187	396	245	326	232

^*#*^ introduced species

* species used in the CCA

**Fig 3:**
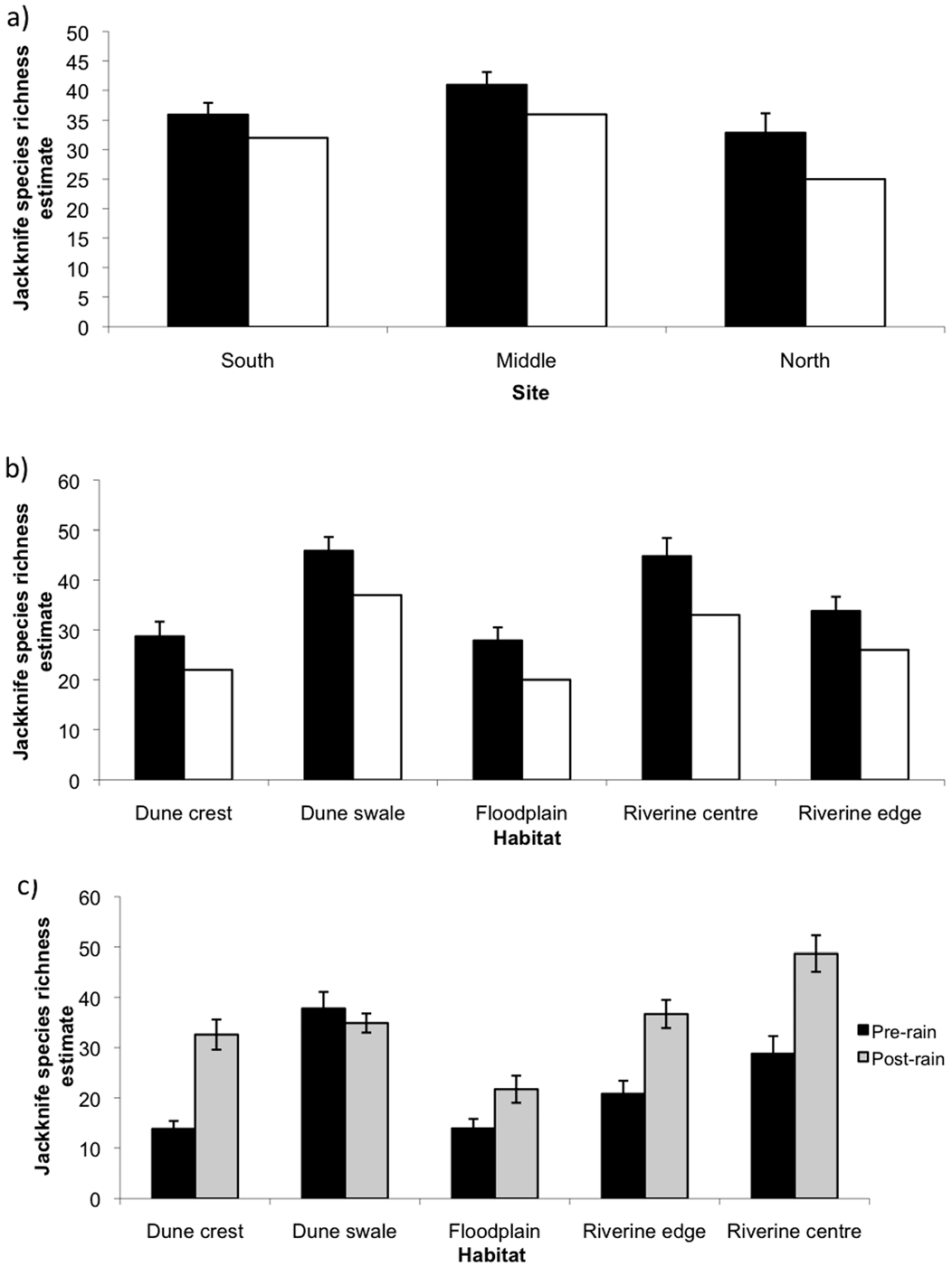
Species richness estimates for small terrestrial vertebrates at a) sites, b) habitats and c) pre- and post-rain in each of five habitats along the Field River, Simpson Desert, western Queensland. Solid bars indicate Jackknife estimates and open bars indicate observed species richness. Error bars indicate standard deviations for Jackknife estimates.

Comparisons of actual species richness with jackknife estimates across sites and habitats indicated that the latter estimates ranged from 6% to 37% higher than simple counts of species (Fig 3). Estimates using both measures were slightly higher in the Field River Middle than in the North or South sites, but differences were not marked (Fig 3a). At the habitat level, dune swales and the riverine centre had the highest estimated species richness, and floodplain the lowest (Fig 3b). Taken together, the dune habitats (dune swale and dune crest) had 38 species (jackknife = 45 ± 3.5 SD) and the riverine habitats (floodplain, riverine centre, riverine edge) had 40 species (jackknife = 54 ± 4.2 SD). Excluding the floodplain habitat, which was present only at Field River North, reduced overall riverine species richness to 39 but did not affect jackknife estimates. In all habitats except the dune swale, estimated species richness increased following the rain in January 2007 (Fig 3c). Increases ranged from 40% in the floodplain to 128% on dune crests (Fig 3c).

### Species composition

Of the 46 species that were detected over the study period, only seven were found exclusively in one habitat (Table 1). The Western Brown Snake (*Pseudonaja nuchalis*) (n = 2) was captured only in the floodplain, Lea’s Ctenotus (*Ctenotus leae*) (n = 2) on the dune crest, the Fat-tailed Gecko (*Diplodactylus conspicillatus*) (n = 6) and Central Bearded Dragon (*Pogona vitticeps*) (n = 2) in the dune swale, Western Hooded Scaly-foot (*Pygopus nigriceps*) (n = 1) in the riverine centre and the Tree Dtella (*Gehyra variegata*) (n = 1) and Burton’s Legless Lizard (*Lialis burtonis*) (n = 2) in the riverine edge. Eight species were found exclusively in the riverine habitats (floodplain, riverine centre, riverine edge), six in the dune habitats (dune swale and crest) and 12 species were ubiquitous, occurring across all habitats (Table 1).

**Fig 4:**
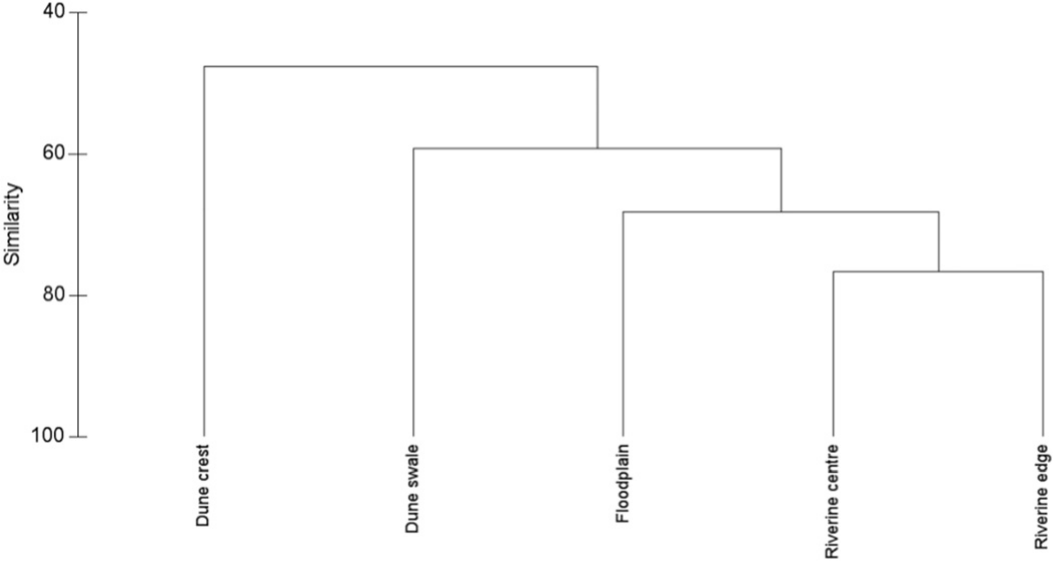
Similarity (%) in species composition of small terrestrial vertebrates (Bray-Curtis similarity index) among five habitat types along the Field River, Simpson Desert, western Queensland.

The Bray-Curtis similarity index revealed that species composition differed among sites and among habitats. The riverine edge and riverine centre had the most similar species composition (76.7%, Fig 4). The greatest dissimilarity in species composition over habitats was between the dune crest and floodplain (43.7%). The SIMPER procedure showed that there was on average 59% similarity in species composition among the habitats. The most abundant species, *Pseudomys hermannsburgensis*, contributed 35.5% to the average similarity among the habitats, while *Lerista labialis* and *Ctenophorus nuchalis* contributed 12.9% and 11.6%, respectively. Sudell's Frog (*Neobatrachus sudellae*), Spade-foot Toad (*Notaden nichollsi*), *Rhynchoedura eyrensis*, *Ctenophorus isolepis*, *Morethia ruficauda*, Helen's Ctenotus (*Ctenotus helenae*), *Mus musculus* and Lesser Hairy-footed Dunnart (*Sminthopsis youngsoni*) together contributed 30%. The 35 remaining species contributed less than 10% to the average similarity among habitats.

**Fig 5:**
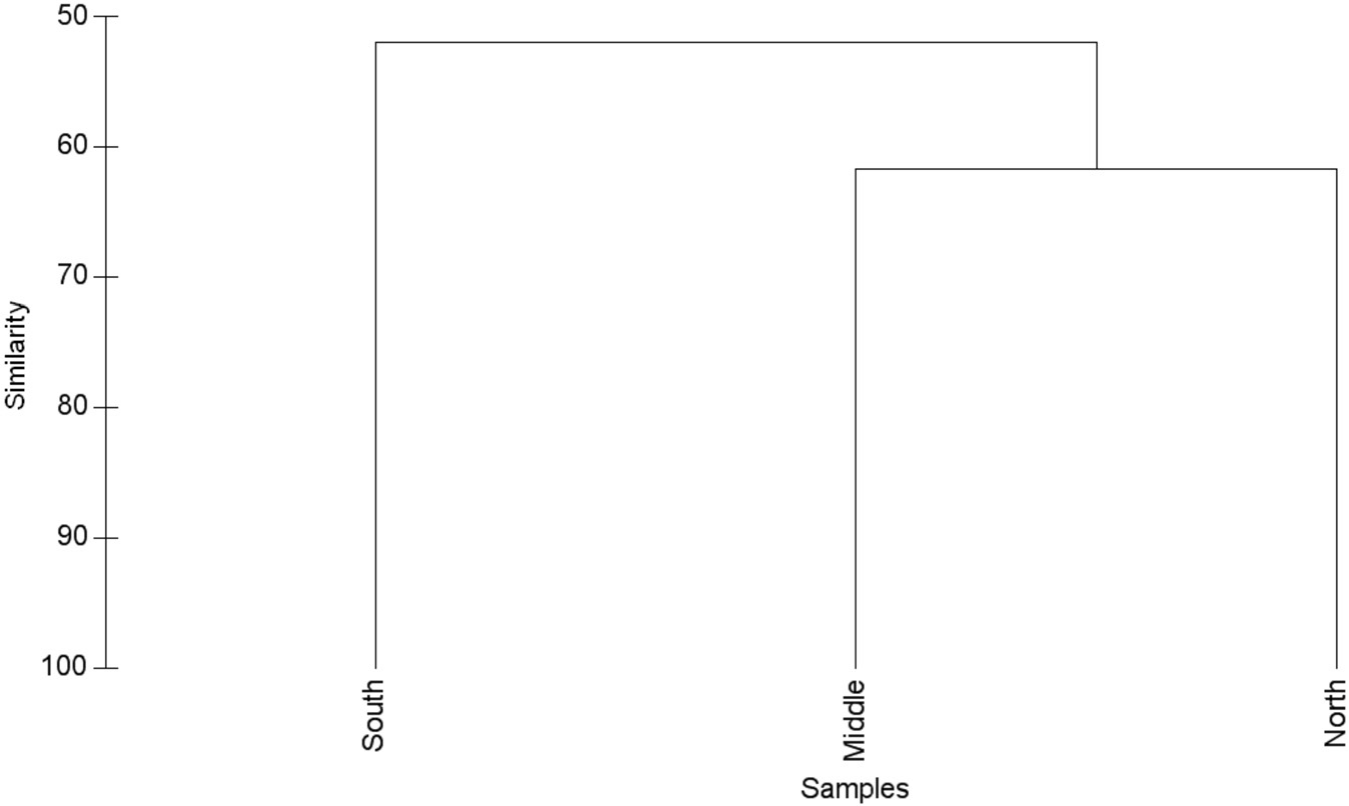
Similarity (%) in species composition of small terrestrial vertebrates (Bray-Curtis index) among three study sites along the Field River, Simpson Desert, western Queensland.

There was moderate similarity (55%) in species composition, on average, among the Field River North, Middle and South sites, although the middle and northern sites were most closely related in regard to vertebrate species composition (Fig 5). As with habitats, the most abundant species (*P*. *hermannsburgensis*, *L*. *labialis*, *C*. *nuchalis*) were the major contributors to between-site similarity.

### Environmental associations

**Fig 6:**
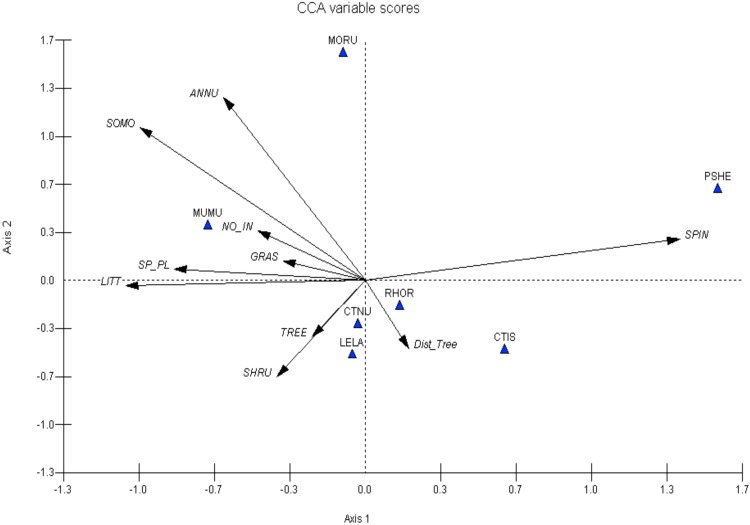
Canonical correspondence analysis (CCA) showing species-environment associations for selected small terrestrial vertebrates in Spring 2006 at the Field River, Simpson Desert, western Queensland. Environmental variables: SPIN = Spinifex cover, Dist_tree = Distance to the nearest tree, SHRU = Shrub cover, TREE = Tree cover, LITT = Litter and detritus cover, SP_PL = Plant species richness, GRAS = Grass cover, NO_IN = Number of invertebrates, SOMO = Soil moisture content, ANNU = Annual cover. Vertebrate species: RHOR = *Rhynchoedura eyrensis*, PSHE = *Pseudomys hermannsburgensis*, CTIS = *Ctenophorus isolepis*, CTNU = *Ctenophorus nuchalis*, LELA = *Lerista labialis*, MUMU = *Mus musculus*, MORU = *Morethia ruficauda*.

In 2006, pre-rain, spinifex cover, cover of annual plants, soil moisture and litter were the most important environmental variables explaining species distributions. *Pseudomys hermannsburgensis* was correlated strongly with spinifex cover, *M*. *musculus* with soil moisture, litter, cover of annual plants, and numbers of invertebrates. In contrast to the mammals, the lizards *Ctenophorus nuchalis*, *C*. *isolepis*, *L*. *labialis*, *M*. *ruficauda* and *R*. *eyrensis* were not strongly correlated with any of the measured variables (Fig 6).

**Fig 7:**
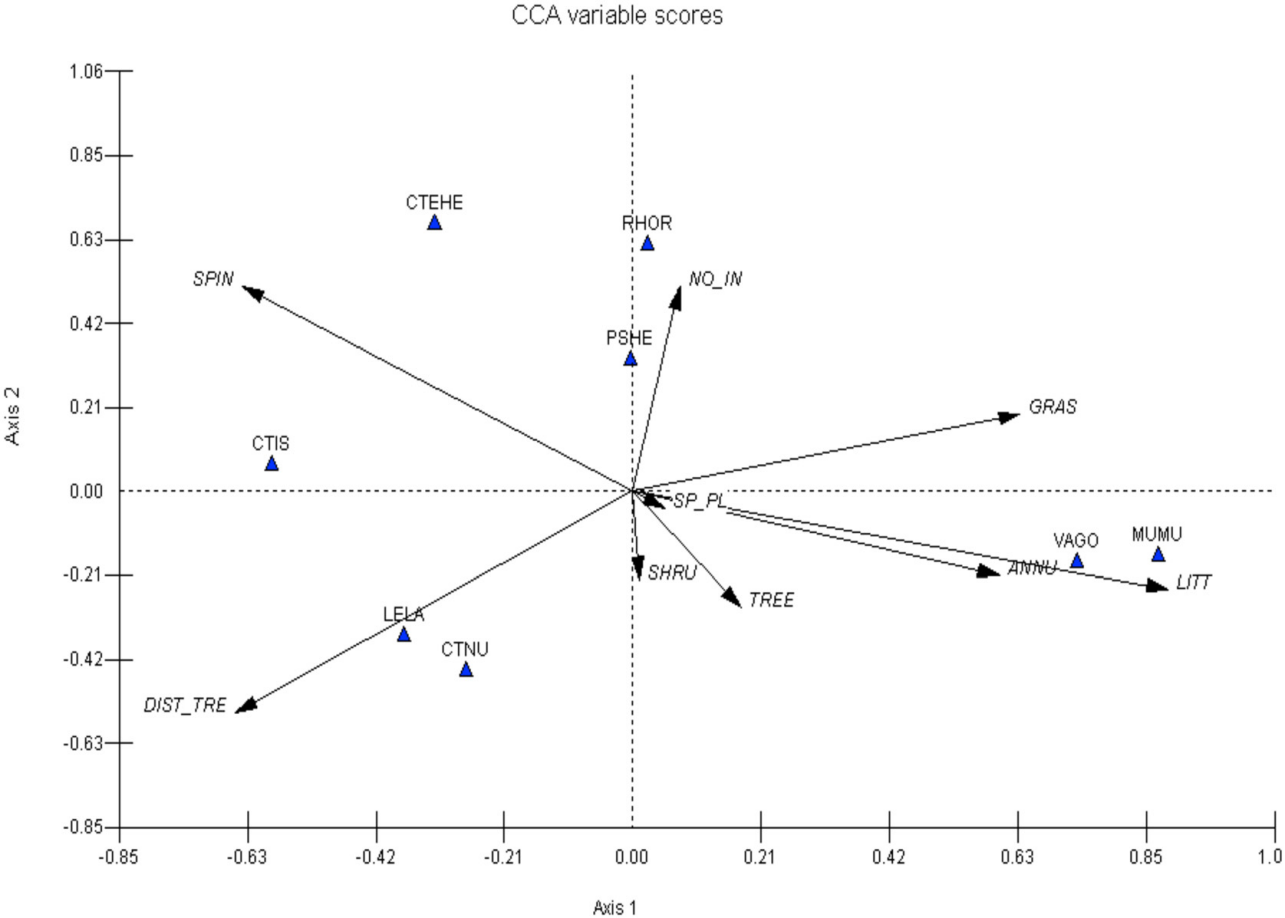
Canonical correspondence analysis (CCA) showing species-environment associations for selected small terrestrial vertebrates in Spring 2007 at the Field River, Simpson Desert, western Queensland. Environmental variables: SPIN = Spinifex cover, DIST_TRE = Distance to the nearest tree, SHRU = Shrub cover, TREE = Tree cover, LITT = Litter and detritus cover, SP_PL = Plant species richness, GRAS = Grass cover, NO_IN = Number of invertebrates, ANNU = Annual cover. Species: RHOR = *Rhynchoedura eyrensis*, PSHE = *Pseudomys hermannsburgensis*, CTIS = *Ctenophorus isolepis*, CTNU = *Ctenophorus nuchalis*, LELA = *Lerista labialis*, MUMU = *Mus musculus* and VAGO = *Varanus gouldii*.

Environment-species associations changed greatly following the heavy rainfall in January 2007. Spinifex cover, cover of annual plants, distance to the nearest tree, grass cover and litter cover were the most important variables explaining species distributions. *Pseudomys hermannsburgensis* became dissociated from spinifex cover and became more strongly correlated with the number of invertebrates; *R*. *eyrensis* also showed a strong association with numbers of invertebrates. *Mus musculus* was still associated highly with the cover of annuals and leaf litter cover. *Ctenophorus nuchalis* and *L*. *labialis* were associated negatively with distance to the nearest tree. The Sand Monitor (*Varanus gouldii*), not shown in 2006 due to small sample size, was associated highly with the cover of leaf litter and annual plants (Fig 7).

## Discussion

Our results provide some support for previous studies [e.g. 4, 7, 37] in showing that riparian areas contain higher levels of vertebrate diversity than their neighbouring habitats, and that riverine and surrounding habitats support some species that are unique to each [38]. Although we found more species and higher estimated levels of species richness in riverine than in dune habitats, the overall difference was modest and driven largely by the difference among the species-rich riverine centre habitats and the relatively impoverished dune crests. Nonetheless, the results are consistent with our general expectation that riparian sites in arid environments support higher vertebrate diversity than other desert habitats, and thus permit us to address the three contingent hypotheses about the processes that generate this pattern of high diversity along desert river systems. We focus our discussion on these below.

### Riverine vertebrate diversity

Our first hypothesis, that sites closer to the edge of the desert would have higher species richness than sites closer to the desert interior, received no support as both actual and estimated species richness were similar in our northern site (closest to the catchment on the desert fringe) and in the southern and middle sites. Three explanations may account for the lack of support for this hypothesis. Firstly, the 40 km between our southern-most and northern-most sample sites may have been insufficient to detect changes in species richness along the riverine corridor. We did not sample the edge of the desert immediately south of the catchment area, and thus may have missed species that extended to the desert fringes. However, this seems unlikely. The 40 km section of river that we did sample shows a very marked gradient of increasing soil moisture, soil nutrients and vegetation cover towards the headwaters on the desert fringe [21]. If peri-desert species do penetrate the desert while exploiting such apparently favourable habitats, our sampling should have uncovered them. Secondly, our sampling sites exhibited differences in species composition, with the middle and northern sites having more species in common than the southern (interior-most) site. This could arise if peri-desert species near the desert fringe are replaced by more desert-adapted species towards the desert interior. However, we found little evidence for this possibility. Most of the species found at the northern but not the southern site, such as the Desert Tree Frog (*Litoria rubella*), Western Brown Snake (*Pseudonaja nuchalis*) and Long-nosed Dragon (*Gowidon longirostris*), have extensive distributions in Australia's interior deserts, whereas some of the species restricted to the southern site, such as Burton's Legless Lizard (*Lialis burtonis*) and the Desert Collared Frog (*Cyclorana cultripes*), have extensive distributions that encompass high-rainfall areas elsewhere [39]. Hence, while species richness was similar across our sampling sites, there was no indication that peri-desert species were replaced by more xeric counterparts along the riverine gradient. Thirdly, and most plausibly, species' occurrences are determined largely by habitat and other environmental factors at the local site level; we explore this possibility more fully below.

Our second hypothesis, that high diversity along the riverine corridor is due to the corridor functioning as a dry-period refuge, also received no support. Indeed, estimated species richness was greatest following heavy rain in all habitats except the dune swale, the converse of the result predicted if this hypothesis were to be supported. If animals were using the riverine habitats as a refuge it could be expected further that species richness following heavy rain would increase in the dune habitats and decrease in the riverine habitats as animals move out of the riverine refuges and into the dune habitats. The general increase in estimated species richness that we observed is, instead, possibly indicative of increased activity by animals following rainfall. The increase in primary productivity in all habitats [21] following the rainfall as well as the initiation of reproduction for many species such as frogs (Table 1) may have led to increased activity and increased likelihood of capture. In addition, and consistent with the results we present here, several recent studies show that small desert-dwelling mammals expand the range of habitats they occupy after rain as conditions across the landscape become temporarily suitable [40–43]; they then continue to exploit their favoured habitats as well as habitats that are not usually used. If these interpretations are correct, the refuge model of Morton [17] may be applicable primarily to medium-sized, relatively mobile mammals which have disappeared from the Field River region [44], rather than to small vertebrates such as those sampled here.

Our results are most consistent with the predictions of the third hypothesis, and indicate that the riverine corridor harbours some species that did not occur in the surrounding dune swale and crest habitats, as well as several species that were ubiquitous across all habitats. Although between-habitat differences in actual and estimated numbers of species were modest, the overall compositional difference in the representation of species was marked (Fig 4), with the riverine habitats clustering distinctly from those of the surrounding sand dunes. Two processes appear likely to drive these patterns: 1) vertebrates may visit the riverine corridor to access higher productivity habitats or resources that are less abundant or not available in the surrounding dunes, and 2) both spinifex-associated species and woodland-associated species utilise the riverine corridor, but woodland-associated species may avoid the spinifex grasslands of the dune habitats.

Firstly, as described in [21] the riverine habitats have greater levels of soil moisture, soil nutrients, grass, tree and annual plant cover than the dune habitats. These characteristics indicate higher productivity and perhaps increased protection from predators and foraging opportunities for vertebrates that are not available in the surrounding dune habitats. Sabo et al. [38] suggested similarly that mobile animals may take advantage of the resources supplied by riparian areas, such as surface water or seasonally favourable microclimates, but still remain dependent on the surrounding landscapes.

Along the Field River, both annual herbs and grasses likely provide food for omnivores such as *Pseudomys hermannsburgensis* which consume both seeds and vegetation [45]. Previous research has shown that some desert rodents, including *P*. *hermannsburgensis*, preferentially select foods that contain high levels of moisture [45]. Plants in the riverine habitats, particularly annuals, contain a higher percentage of water in their structures due to the high availability of water in the soil [46]. This may be advantageous to desert-dwelling herbivores and omnivores that rely on the water they obtain through their diets. Similarly, plants in the riverine habitats may have higher levels of nitrogen in their structures due to elevated levels of N in the soil [46] and, as nitrogen is a limiting nutrient in arid Australia [47], these plants are probably important dietary items.

Temporary increases in food or habitat resources along the riverine corridor may also lead to short term visits by mobile spinifex-associated species, increasing species richness temporarily. For example, following local rain and the growth of Annual Verbine (*Psoralea cinerea*) there was a noticeable increase in the number of grasshoppers in the riverine areas where the plant grows (C. Free, pers. obs.). This localised resource pulse may lead to temporary increases in abundance of mobile omnivorous/insectivorous species such as *Sminthopsis youngsoni* which can travel up to 12 km to access resources [48]. Other insectivorous mammals, reptiles and birds may immigrate to the riverine corridor temporarily in order to access such resources and thereby increase the diversity in the short term.

Secondly, species richness may be greater in the riverine centre habitats due to the combination of species that rely on the river as well as ubiquitous species that inhabit both the riverine habitats and spinifex-dominated dune habitats. Compositional analysis showed that the species complement was most similar in the riverine edge and centre habitats, with species such as the Desert Burrowing Frog (*Platyplectrum spenceri*), *Litoria rubella*, *Gehyra variegata*, *Gowidon longirostris*, *Lialis burtonis*, *Pygopus nigriceps* and the Excitable Delma (*Delma tincta*) found only in the riverine habitats. Although these species have extensive distributions in inland Australia, *G*. *longirostris*, *G*. *variegata* and *L*. *rubella* prefer habitats with trees or access to water, while the remaining species forage or shelter in leaf litter and detritus [49, 50]. All these material resources are more abundant and accessible in the riverine habitats than in the dunes [21], with the greater amounts of leaf litter and other organic matter generated by the trees and grasses. Trees, fallen limbs and branches may also provide shelter and foraging opportunities for arboreal species such as the Pygmy Mulga Monitor (*Varanus gilleni*) that were captured largely but not exclusively in riverine habitat.

Species composition was most different between the riverine habitats and the dune crest, with species such as the ctenotuses *Ctenotus ariadnae*, *C*. *dux*, *C*. *leae* and *C*. *calurus* captured only in dune habitats (Table 1). These species are considered sandridge or spinifex specialists [50, 51]. The riverine corridor had very little spinifex cover [21] and therefore was unlikely to meet the habitat requirements of these species. Despite the relatively close proximity of the riverine habitats to the sand dunes, the restricted patterns of species occurrence that we found here are reflected in other studies that have been carried out in the broader sand dune environment away from riparian areas. Thus, the ctenotuses that we found have been recorded in sand dune habitat east and north-east of the Field River many kilometres from any riparian or lacustrine habitats [52], as well as further west in open sand desert habitats [39, 51], suggesting very limited or no proximity effect of riverine habitat on species' distributions.

Many (12 of 46) of the species recorded during this study were ubiquitous in all habitats. *Lerista labialis*, *Ctenophorus isolepis*, *C*. *nuchalis* and *Pseudomys hermannsburgensis* are usually associated with spinifex grassland [50, 53] but were recorded frequently along the riverine corridor. These species may not necessarily rely on the corridor but may utilise the resources along it or use it during various stages throughout their life cycle.

This combination of ubiquitous species and those that only or largely inhabit the riverine corridor has increased the local diversity of the Field River and could indicate the river is a focal point for diversity in the broader area. However, in order to test this possibility, comparisons of diversity will need to be made on a much larger scale in other desert river systems.

### Species-environment correlations

The results of the species-environment analyses allow us to further explore the broad patterns of habitat occupancy discussed above. Two species of mammal were captured sufficiently often to assess their habitat use before and after the heavy rain of January 2007. The first, *M*. *musculus*, was associated with the cover of leaf litter and annual plants both before and after the rainfall event. Strong associations between this species with numbers of invertebrates and soil moisture occurred prior to the rain; unfortunately soil moisture was not measured after the rain, but the association with invertebrates disappeared. The consistent correlation of *M*. *musculus* with leaf litter and annuals and with soil moisture pre-rain suggests that this species' distribution in the desert may be limited at least partly to areas with high soil moisture and persistent resource availability. In both sampling sessions, the distribution of *M*. *musculus* was inversely related to spinifex cover, consistent with the idea that *M*. *musculus* is a poor coloniser of spinifex grassland [54]. *Mus musculus* and *P*. *hermannsburgensis* were also found to lie on opposite sides of the biplot, especially pre-rain (Figs 6 and 7), suggesting an inverse relationship. This may suggest that *P*. *hermannsburgensis* is able to outcompete or limit the distribution of *M*. *musculus* [47].

*Pseudomys hermannsburgensis* was very strongly correlated with spinifex cover before the rain, which supports suggestions [55] that this species prefers habitats with high cover. However, following the rain, animals became more associated with the number of invertebrates captured. *Pseudomys hermannsburgensis* is an omnivore that consumes seeds, vegetation and invertebrates as part of its diet [56], and it is plausible that animals changed their diet to take advantage of the post-rain flush in invertebrate abundance [21], perhaps even at the expense of *M*. *musculus*. In cafeteria trials, Murray and Dickman [45] found that *P*. *hermannsburgensis* preferentially consumes invertebrates over seeds, plant stems and fungus. Insects are high in protein (nitrogen) and water [45] and this may be beneficial to this rodent during breeding when protein requirements are high. *Mus musculus* fed on low-protein diets in the laboratory have decreased breeding performance and delayed sexual maturity [57]. Further, nitrogen is considered to be a limiting factor in arid Australia [41] and the protein available in invertebrates may provide the nutrients necessary for breeding.

Prior to the rainfall event in January 2007 there was little indication that reptile species were associated with any of the environmental variables that we measured, perhaps indicating their broad distribution among habitats. After the rain, associations became more apparent. *Ctenophorus nuchalis* and *Lerista labialis* were most strongly associated with increasing distances to trees. This result is not surprising for the fossorial *L*. *labialis*, which prefers non-compacted soft soil usually associated with dune crests [58]. On the other hand, *C*. *nuchalis* perches on fallen tree branches, trunks of trees and shrubs for thermoregulatory purposes [59, 60]; these components occur mostly in association with the riverine habitats. The shift away from trees post-rain may reflect increased usage by *C*. *nuchalis* of open habitats to exploit higher temperatures away from cover [59, 60], or possibly to reduce the risk of encounters with the predatory *Varanus gouldii* (see Fig 7). A further association to emerge post-rain was that of *Rhynchoedura eyrensis* with numbers of invertebrates. Species in this genus are insectivorous and may specialise on termites [61], although the diet of *R*. *eyrensis* has not been studied. If *R*. *eyrensis* includes termites in its diet, it is possible that this gecko was tracking increased termite activity that would be expected in moister conditions post-rain. Food availability appears to be a key driver of desert lizard assemblages [52]. We caution that these interpretations are based on relatively small sample sizes on a subset of species in the desert study system, and that longer-term studies are needed to arrive at firmer conclusions.

## Conclusions and Implications for Conservation

The results of this study suggest that, at least on a local scale, vertebrate diversity is greatest along the Field River corridor. If other desert river habitats in Australia harbour species-rich assemblages or species that do not occur in the broader landscape [5, 8, 9], as they do in other world desert systems [7, 38], these linear features should be seen as priorities for conservation. Although long neglected, our results suggest that desert river habitats may be important repositories for wildlife. Focus on riverine habitats does not in any way diminish the importance of habitats in the surrounding sand dune environment, but simply acknowledges that desert rivers occupy tiny fractions of the overall landscape compared with the open desert. We recommend that further surveys of inland river systems be carried out to identify those of highest conservation value, and management initiated where possible to alleviate threats to their continued functioning.
